# Clinical correlates and prognostic implications of severe suicidal ideation in major depressive disorder

**DOI:** 10.1097/YIC.0000000000000461

**Published:** 2023-02-27

**Authors:** Paolo Olgiati, Giuseppe Fanelli, Alessandro Serretti

**Affiliations:** a Department of Biomedical and Neuromotor Sciences, University of Bologna, Bologna, Italy; b Department of Human Genetics, Radboud University Medical Center, Donders Institute for Brain, Cognition and Behaviour, Nijmegen, The Netherlands

**Keywords:** bipolar disorder, bipolar features, child abuse, childhood maltreatment, early life stress, insomnia, major depression, suicide attempt, suicidal behaviour, suicidal thoughts

## Abstract

Suicidal ideation (SI) is a risk factor for suicidal behaviour. To ascertain the clinical correlates and prognostic impact of severe SI, we analysed 249 outpatients with major depressive disorder (MDD) and suicidal thoughts included in the COmbining Medications to Enhance Depression outcome (CO-MED) trial. Patients with severe SI (36%) were younger at disease onset (*P* = 0.0033), more severely depressed (*P* = 0.0029), had more lifetime suicidal behaviour (*P* < 0.0001) and psychiatric comorbidities (panic disorder: *P* = 0.0025; post-traumatic stress disorder: *P* = 0.0216), and a history of childhood maltreatment (neglect: *P* = 0.0054; emotional abuse: *P* = 0.0230; physical abuse: *P* = 0.0076; sexual abuse: *P* = 0.0016) than those experiencing low-moderate SI. After controlling for depression score, severe SI was positively correlated with lifetime suicidal behaviour (OR [95% CI]: 1.26 [1.12–1.41]), panic disorder (1.05 [1.00–1.12]), and childhood maltreatment (neglect: 1.93 [1.13–3.30]; physical abuse: 2.00 [1.11–3.69]; sexual abuse: 2.13 [1.17–3.88]), and inversely correlated with age of onset (0.97 [0.95–0.99]) and sleep-onset insomnia (0.76 [0.61–0.96]). Finally, the occurrence of serious lifetime suicidal behaviour was predicted by SI severity (2.18 [1.11-4.27]), bipolar score (1.36 [1.02–1.81]), and childhood sexual abuse (2.35 [1.09–5.05]). These results emphasise the importance of assessing childhood maltreatment and bipolar liability in MDD to estimate suicidal behaviour risk.

## Introduction

Almost 800 000 people (9/100 000) across the world die by suicide every year (Ilic and Ilic, 2022). Although suicide is not merely a psychiatric phenomenon, the great majority of suicide deaths are related to mental illness (Bachmann, 2018). In particular, individuals with major depressive disorder (MDD) have been associated with more than 20 times higher mortality from suicide in comparison with the general population (Osby *et al*., 2001) and an estimated lifetime risk ranging from 2 to 8% (Blair-West *et al*., 1999; Bostwick and Pankratz, 2000; Nordentoft *et al.*, 2011; Hogberg *et al*., 2015). Among depressive symptoms, suicidal ideation (SI), which has been reported in up to 60% of outpatients with MDD (Sokero *et al.*, 2003; Trivedi *et al.*, 2013), is considered to be a precursor of completed suicide. According to a meta-analysis, suicide was 2.3 times more likely in patients with mood disorders who had previously expressed SI than in their counterparts who had not (Hubers *et al*., 2018). Research has indicated several factors increasing the risk of SI in depressive disorders. Among them, a pivotal role is played by clinical variables such as depression severity, age at onset and course of depression (Zisook *et al*., 2011), abuse and violence in childhood (Chou, 2012; Zisook *et al*., 2022) and mental disorder comorbidities (Hardt *et al.*, 2015; Wiebenga *et al*., 2021). Moreover, SI has been related to depression psychopathology, in particular to hopelessness (Wolfe *et al*., 2017; Ribeiro *et al*., 2018; Wiebenga *et al*., 2021) and self-blame (Olgiati *et al*., 2006), irritability (Jha *et al.*, 2020), insomnia (Pigeon *et al*., 2012; Wang *et al.*, 2019) and bipolar features (Akiskal *et al*., 2005; Weinstock *et al*., 2016; Rihmer and Rihmer, 2019). Despite these known risk variables, there is still a knowledge gap in our ability to predict SI and its progression to suicide attempt (SA). One reason could be the definition of SI itself, which is broad and gathers thoughts at different levels of dangerousness, from a generic wish to die to a detailed plan to end one’s life (House *et al*., 2020). If common sense suggests a relationship between SI and SA severity, research is only beginning to unravel the intrinsic heterogeneity of SI and its impact on suicidality. In a series of studies performed to validate the Columbia-Suicide Severity Rating Scale (C-SSRS), individuals with the highest levels of SI were found to be at increased risk for attempting suicide (Posner *et al*., 2011; Greist *et al*., 2014). Another work suggested a correlation between SI severity and more serious SAs carried out with lethal methods (Shelef *et al*., 2019). Little is known about the presence of different predictors for severe and mild SI. We are aware of only one study conducted in Spain, in which callers to a telephone helpline were subdivided into individuals with low-moderate and severe SI. Callers with severe SI differed from those with low-moderate SI in being younger than 50 years, having a greater attempt capability, greater hopelessness and lack of life sense, as well as previous SA and preparatory acts (Fernández-Montalvo *et al*., 2021).

The present study aims to confirm the association between SI severity and suicidal behaviour by controlling for a number of potential confounding variables, such as depression severity (Zisook *et al*., 2011), childhood maltreatment (Chou, 2012; Zisook *et al*., 2022) and bipolar liability (Park, 2017). We also aim to test a pool of variables that have consistently been associated with the presence of SI (see Akiskal *et al*., 2005; Zisook *et al*., 2011, 2022; Chou, 2012; Hardt *et al*., 2015; Weinstock *et al*., 2016; Rihmer and Rihmer, 2019; Jha *et al*., 2020; Wiebenga *et al*., 2021) as moderators of its severity degree.

## Methods

### Study sample and design

This study was based on the COmbining Medications to Enhance Depression outcome (CO-MED) trial. Eligible subjects were aged 18–75 years, with a primary DSM-IV diagnosis of nonpsychotic MDD and a HAM-D_17_ score of at least 16 before treatment initiation (Rush *et al.*, 2011). To be included in the current analysis, a further criterion was to report SI during baseline assessment (see below). A single-blind placebo-controlled design was used in the CO-MED trial to compare three treatment arms: (1) escitalopram plus placebo; (2) bupropion SR plus escitalopram; (3) venlafaxine XR plus mirtazapine. The trial included a short-term (12 weeks) treatment followed by a continuation phase (weeks 12–28) (Rush *et al.*, 2011).

The CO-MED trial was conducted according to the Principles of the Helsinki Declaration and its protocol was approved by ethical committees at local recruitment sites. All subjects who met the selection criteria were included in the CO-MED trial after obtaining their written informed consent. This research group certifies that data collected from the CO-MED trial were exclusively used for scientific investigation and, before obtaining access to them, the objectives of our investigation were clearly reported in the request form (Olgiati and Serretti, 2022a).

### Assessment procedure

Research data were collected by a variety of assessment tools as reported in our previous publications (Serretti *et al.*, 2021; Olgiati and Serretti, 2022a; Olgiati *et al*., 2022): (1) a socio-demographic form including age, sex, ethnic group, education and monthly income; (2) the Mini International Neuropsychiatric Interview (MINI) (Sheehan *et al.*, 1998) for diagnostic assessment and to perform a retrospective evaluation of depressive disorder (chronic or recurrent course of depression; number of depressive episodes and age at onset of first episode) and ascertain the lifetime occurrence of subthreshold hypomanic episodes; (3) a battery of scales for cross-sectional clinical assessment including the 30-item Inventory of Depressive Symptomatology-Clinician Rating (IDS-C_30_) (Corruble *et al*., 1999) and the 16-item Quick Inventory of Depressive Symptomatology (QIDS-C16) (Rush *et al.*, 2003), the Concise Associated Symptoms Tracking (CAST) for irritability (Trivedi *et al*., 2011b), the Altman Self-Rating Mania Scale (ASRM) (Altman, 1998) and the Work and Social Adjustment Scale (WSAS) (Mundt *et al.*, 2002) to ascertain functional impairment; (4) the Psychiatric Diagnostic Screening Questionnaire to investigate comorbid mental disorders including panic disorder, generalised anxiety, obsessive-compulsive disorder, social phobia, post-traumatic stress disorder (PTSD) and alcohol and substance use disorders (Zimmerman and Mattia, 1999) and (5) two questionnaires that were specifically developed for the CO-MED project to investigate lifetime suicidal behaviour and experiences of maltreatment during childhood (childhood maltreatment: neglect; emotional abuse; physical abuse and sexual abuse) (Serretti *et al*., 2021; Olgiati and Serretti, 2022a).

### Suicidality-related variables

SI was assessed by means of the Concise Health Risk Tracking-Self Report (CHRT-SR) scale (Trivedi *et al*., 2011a). This scale includes 12 items (overall score: 0–48), of which nine explore a generic suicidal propensity. Our analysis was based on the last three items, which specifically investigate SI and plans over the last 24 h: scoring ≥1 at baseline was used as an inclusion criterion (see paragraph ‘*Study sample and design*’), whereas a threshold ≤2 was selected to define SI remission at week 6. The stratification of SI was based on a cutoff score of 5, which allowed us to differentiate between low-moderate (CHRT-SR: 1–4) and severe (CHRT-SR ≥5) SI groups.

Lifetime suicidal behaviour was rated as follows: 1 = no suicidal tendency, 2 = thoughts of dying, no suicidal thoughts, 3 = occasional thoughts of suicide, no plans; 4 = often thought about suicide or has thought of a specific method; 5 = had a plan or made a gesture; 6 = made preparation for a potentially serious SA; 7 = SA with definite intent to die or be potentially medically harmful. Scores ≥5 were used to define severe suicidal behaviour.

### Statistical analyses

A number of variables were compared between patients who showed low-moderate and severe SI. These included: (1) Socio-demographic variables; (2) chronic depression (episode lasting for at least 6 months); (3) depression severity, including the IDS-C_30_ total score, work and social impairment, and symptom profile, as subdivided into core depressive manifestations (negative self-outlook; negative outlook of future; loss of pleasure; slowing and poor concentration), anxious depression manifestations (anxious mood; irritability and psychomotor agitation) and insomnia (sleep-onset insomnia; middle nocturnal insomnia and early awakening); (4) bipolar spectrum variables, including: (a) age of onset (Benazzi, 2009); (b) mood disorder recurrence (number of episodes/illness years) (Mazzarini *et al*., 2018); (c) hypomanic symptoms occurring within the major depressive episode (ASRM items): cheerfulness; self-confidence; reduced need for sleep; talkativeness and goal-oriented hyperactivity; (d) mixed depression, defined as a major depressive episode with three or more concurrent hypomanic symptoms (Akiskal *et al.*, 2005); (e) lifetime subthreshold hypomania: a period of elated or irritable mood with at least two concurrent hypomanic symptoms (MINI interview), which did not fulfil DSM criteria for hypomanic/manic episode (Angst *et al*., 2003; Serretti *et al.*, 2021). Finally, a bipolar spectrum score was calculated as follows: age of onset <21: 1 point; mixed depression: 2 points; subthreshold hypomanic episode: 2 points; (5) comorbid mental disorders; (6) childhood maltreatment; (7) prognostic variables including remission of depressive symptoms (IDS-C_30_ <7) and SI (CHRT-SR ≤2) during acute treatment (week 6) and lifetime suicidal behaviour. The most clinically meaningful comparisons (bipolar features; childhood maltreatment; psychiatric comorbidities and lifetime suicidal behaviour) were replicated in a smaller sub-sample with mild depressive symptomatology (IDS-C ≤35).

Univariate analyses were performed using Student’s *t*-test for continuous variables and the Mantel-Haenszel chi-square test to analyse linear trends between SI levels. Multiple logistic regression analysis was used to test the association of each variable with SI severity by controlling for overall depression score, the main confounding factor correlated with SI, and to perform multivariate analyses of lifetime suicidal behaviour.

A preliminary power analysis was conducted to estimate minimum detectable differences between comparison groups, considering a type I error (alpha level) of 0.05 and a type II error (1-power) of 0.20 (Abraham and Russell, 2008). Power analysis was carried out via G*Power 3 (Faul *et al.*, 2007).

The significance threshold was set at alpha = 0.025, with a minimal correction for multiple testing as discussed elsewhere (Amrhein *et al*., 2019). All the analyses were conducted in the OpenStat version December 8, 2014 (https://openstat.info/OpenStatMain.htm).

## Results

The study sample consisted of 249 outpatients with MDD and SI. Their mean age was 43.04 ± 12.51 years; 76 patients (31%) were males and 164 (66%) were of Caucasian origin. The overall depression score (IDS-C_30_) was equal to 40.56 ± 9.36 at treatment start and decreased to 22.54 ± 12.12 after 6 weeks of antidepressant use (*t* = 17.77; *P* < 0.0001), whereas the SI score varied from 4.00 ± 2.31 at baseline to 1.75 ± 2.25 at week 6 (*t* = 10.38; *P* < 0.0001).

### Power analysis

The sample, which included 159 patients with low-moderate SI and 90 severe SI subjects, was adequately powered (0.80) to detect small-medium effect sizes (*d* = 0.369) or differences of at least 8% between comparison groups.

### Demographic characteristics, depression features and bipolar spectrum variables

The distribution of socio-demographic characteristics, depressive symptoms and bipolar spectrum variables are reported in Table 1.

**Table 1:** Distribution of socio-demographic and symptomatological variables between patients with major depressive disorder experiencing low-to-moderate and severe suicidal ideation.

	Low-moderate SI	Severe SI	*P* value
	(*N* = 159)	(*N* = 90)	
Age	44.18 ± 12.98	41.03 ± 11.44	0.0568
Work/social impairment	27.52 ± 8.27	30.60 ± 8.37	**0.0213**
Depression symptoms			
IDS-c_30_ (total score)	39.24 ± 9.27	42.90 ± 9.11	**0.0029**
Negative self-outlook	1.94 ± 0.87	2.17 ± 0.85	0.0516
Negative outlook of future	1.64 ± 0.87	1.86 ± 0.82	0.0583
Loss of pleasure	1.54 ± 0.88	1.77 ± 0.92	0.0568
Anxious mood	1.84 ± 0.78	1.98 ± 0.76	0.1888
Difficulty in falling asleep	1.94 ± 1.22	1.79 ± 1.34	0.3567
Middle nocturnal insomnia	2.04 ± 1.10	2.20 ± 1.06	0.2788
Hypomanic symptoms			
More cheerful	0.28 ± 0.66	0.18 ± 0.53	0.2228
More self-confident	0.30 ± 0.71	0.21 ± 0.55	0.3295
Reduced need for sleep	0.53 ± 1.00	0.81 ± 1.34	0.0664
More active	0.18 ± 0.57	0.11 ± 0.55	0.3391
Bipolar spectrum variables			
Age of onset (first episode)	24.86 ± 14.01	19.47 ± 13.36	**0.0033**
Recurrence (episodes/year)	0.32 ± 0.55	0.51 ± 1.25	0.1085
Mixed depression	26 (0.16)	10 (0.11)	0.2505
Subthreshold hypomania	14 (0.09)	14 (0.16)	0.1060
Bipolar score	1.01 ± 1.14	1.26 ± 1.13	0.1064

Significant differences (*P* < 0.025) are in bold.

Only variables with *P* < 0.40 at univariate analyses are reported.

Values are means ± SD or *N* (%).

IDS-C_30_, 30-item Inventory of Depressive Symptomatology-Clinician Rating; SI, suicidal ideation.

Patients with severe SI were marginally younger (*P* = 0.058), and more severely depressed (IDS-C_30_ total score: *P* = 0.0029; work and social impairment: *P* = 0.0213) than their counterparts with low-moderate SI (Table 1). Although no depressive symptom was associated with SI severity at the univariate level, after controlling for depression severity there was a negative correlation between difficulty in falling asleep and SI severity [odds ratio (OR), 0.76; 95% confidence interval (CI), 0.61–0.96] (Table 2).

**Table 2:** Association of socio-demographic characteristics, depressive and hypomanic symptoms, and bipolar spectrum variables with suicidal ideation severity

Predictors	OR	95% CI
Difficulty in falling asleep	**0.76**	**0.61–0.96**
Age of onset	**0.97**	**0.95–0.99**

Significant associations (*P* < 0.025) are in bold.

Only significant suicidal ideation predictors are reported.

Depression severity (IDS-C_30_ total score) was included as a covariate.

CI, confidence intervals; IDS-C_30_, 30-item Inventory of Depressive Symptomatology-Clinician Rating; OR, odds ratio.

With regard to the bipolar spectrum variables, age of onset was the only one associated with SI severity at the univariate level (Table 1). Indeed, patients who reported severe SI were younger during their first depressive episode compared to those who had low-moderate SI (*P* = 0.0033). This result was confirmed after controlling for depression severity (OR, 0.97; 95% CI, 0.95–0.99) (Table 2).

### Psychiatric comorbidities and childhood maltreatment

Patients with MDD experiencing severe SI were associated with panic disorder (*P* = 0.0025) and PTSD (*P* = 0.0216) comorbidities, as well as with childhood maltreatment (i.e., a larger number of events) (1.88 vs. 1.21; *P* = 0.0004), neglect (51/90 vs. 61/159; *P* = 0.0054), emotional abuse (51/90 vs. 68/159; *P* = 0.023), physical abuse (32/90 vs. 32/159; *P* = 0.0076) and sexual abuse (34/90 vs. 31/159; *P* = 0.0016) (Table 3). Panic disorder (OR, 1.05; 95% CI, 1.00–1.12) and childhood maltreatment (number of maltreatment events: OR, 1.32; 95% CI, 1.09–1.59; neglect: OR, 1.93; 95% CI, 1.13–3.30; physical abuse: OR, 2.00; 95% CI, 1.11–3.63; sexual abuse: OR, 2.13; 95% CI, 1.17–3.88) were confirmed to be predictors of SI severity after controlling for baseline depression severity (Table 4).

**Table 3:** Distribution of psychiatric comorbidities, and childhood maltreatment variables between patients with major depressive disorder experiencing low-moderate and severe suicidal ideation

	Low-moderate SI	Severe SI	*P* value
	(*N* = 159)	(*N* = 90)	
Comorbidities (PDSQ scores)			
Panic disorder	4.30 ± 4.76	6.33 ± 5.52	**0.0025**
GAD	6.82 ± 2.73	7.50 ± 2.83	0.0649
PTSD	3.50 ± 4.08	4.80 ± 4.54	**0.0216**
Binge-eating	2.47 ± 3.09	2.63 ± 3.41	0.7028
OCD	1.28 ± 1.89	1.49 ± 1.97	0.4176
Social phobia	5.27 ± 5.10	6.72 ± 5.20	0.0330
Alcohol	0.67 ± 1.48	0.71 ± 1.48	0.8199
Substance	0.30 ± 1.03	0.37 ± 1.18	0.6510
Childhood maltreatment			
Number of maltreatment events	1.21 ± 1.32	1.88 ± 1.57	**0.0004**
Neglect	61 (0.38)	51 (0.57)	**0.0054**
Emotional abuse	68 (0.43)	52 (0.58)	**0.0230**
Physical abuse	32 (0.20)	32 (0.35)	**0.0076**
Sexual abuse	31 (0.19)	34 (0.37)	**0.0016**

Significant differences (*P* < 0.025) are in bold.

Values are means ± standard deviations or N (%).

GAD, generalised anxiety order; OCD, obsessive-compulsive disorder, PDSQ, Psychiatric Diagnostic Screening Questionnaire; PTSD, post-traumatic stress disorder; SI, suicidal ideation.

**Table 4:** Association of psychiatric comorbidities, and childhood maltreatment variables with suicidal ideation severity

Predictors	OR	95% CI
Panic disorder	**1.05**	**1.00–1.12**
GAD	1.04	0.94**–**1.16
PTSD	1.06	0.99**–**1.12
Binge-eating	1.00	0.92**–**1.08
OCD	1.04	0.90**–**1.18
Social phobia	1.04	0.98**–**1.09
Alcohol	1.05	0.88**–**1.25
Substance	1.05	0.83**–**1.33
Number of maltreatment events	**1.32**	**1.09–1.59**
Neglect	**1.93**	**1.13–3.30**
Emotional abuse	1.61	0.94**–**2.76
Physical abuse	**2.00**	**1.11–3.63**
Sexual abuse	**2.13**	**1.17–3.88**

Significant associations (*P* < 0.025) are in bold.

Depression severity (IDS-C_30_ total score) was included as a covariate.

CI, confidence intervals; GAD, generalised anxiety disorder; IDS-C_30_, 30-item Inventory of Depressive Symptomatology-Clinician Rating; OCD, obsessive-compulsive disorder, OR, odds ratio, PTSD, post-traumatic stress disorder, SI, suicidal ideation.

### Suicidal ideation severity in patient with milder depressive symptomatology

Out of 78 patients characterised by mild depressive symptomatology (IDS-C ≤35), 21 subjects (27%) met the criteria for severe SI; when compared to the rest of the sample, this group was associated with a higher bipolar score (*P* = 0.0118) and more childhood maltreatment (number of maltreatment events, *P* = 0.0088; neglect: *P* = 0.0159; physical abuse: *P* = 0.0204) (Table 5).

**Table 5:** Distribution of depressive severity, psychiatric comorbidities, bipolar spectrum variables, childhood maltreatment variables, and lifetime suicidal behaviour between patients with milder major depressive symptomatology experiencing low-moderate and severe suicidal ideation

	Low-moderate SI	Severe SI	*P* value
	(*N* = 57)	(*N* = 21)	
Depression severity			
IDS-C_30_	29.23 ± 4.36	31.10 ± 3.16	0.0771
Work/social adjustment	23.12 ± 8.42	27.14 ± 9.58	0.0755
Psychiatric comorbidities			
Panic disorder	2.33 ± 3.69	4.33 ± 5.17	0.0681
GAD	5.54 ± 2.90	6.38 ± 3.32	0.2810
OCD	1.02 ± 1.79	1.95 ± 2.22	0.0593
PTSD	2.89 ± 3.86	4.57 ± 4.59	0.1101
Binge-eating	1.86 ± 2.81	2.52 ± 2.96	0.3637
Social phobia	3.35 ± 4.16	5.86 ± 5.15	0.0300
Bipolar spectrum variables^a^			
Age of onset	26.81 ± 13.79	20.67 ± 11.91	0.0750
Bipolar score	0.75 ± 0.95	1.48 ± 1.29	**0.0118**
Childhood maltreatment			
Number of events	0.89 ± 1.21	1.76 ± 1.41	**0.0088**
Neglect	18 (0.31)	13 (0.62)	**0.0159**
Emotional abuse	19 (0.33)	10 (0.48)	0.2499
Physical abuse	8 (0.14)	8 (0.38)	**0.0204**
Sexual abuse	6 (0.10)	6 (0.28)	0.0516
Lifetime suicidal behaviour			
LSB score	1.51 ± 1.78	2.60 ± 2.11	0.0289

Significant differences (*P* < 0.025) are in bold.

Values are means ± standard deviations or N (%).

GAD, generalised anxiety disorder; IDS-C_30_, 30-item Inventory of Depressive Symptomatology-Clinician Rating; LSB, lifetime suicidal behaviour; OCD, obsessive-compulsive disorder; OR, odds ratio; PTSD, post-traumatic stress disorder; SI, suicidal ideation.

a Mixed depression and subthreshold hypomania were not reported because expected values were<5.

### Prognostic role of suicidal ideation, childhood maltreatment and bipolar spectrum variables

In comparison with the low-moderate SI group, patients with MDD experiencing severe SI showed higher lifetime suicidal behaviour scores (*P* < 0.0001), more severe lifetime suicidal behaviour (*P* = 0.0014), and a lower likelihood of achieving SI remission (*P* = 0.0031) (Table 6). All these associations were replicated after controlling for depression severity (lifetime suicidal behaviour score: OR, 1.26; 95% CI, 1.12–1.41; serious lifetime suicidal behaviour: OR, 2.45; 95% CI, 1.29–2.66; SI remission: OR, 0.43; 95% CI, 1.29–4.66) (Table 7).

**Table 6:** Prognostic variables: lifetime suicidal behaviour, depression remission (week 6), and suicidal ideation remission (week 6)

	Low-moderate SI	Severe SI	*P* value
	(*N* = 156)	(*N* = 87)	
LSB score^a^	2.03 ± 2.22	3.59 ± 2.39	**<0.0001**
Severe LSB^a^ (scores 5-7)	23 (0.15)	28 (0.32)	**0.0014**

	Low-moderate SI	Severe SI	*P* value
	(*N* = 124)	(*N* = 74)	
Depression remission	36 (0.29)	13 (0.18)	0.0712
SI remission	85 (0.69)	35 (0.47)	**0.0031**

Significant differences (*P* < 0.025) are in bold.

LSB, lifetime suicidal behaviour; SI, suicidal ideation.

a LSB score: from 1 = no suicidal tendency to 7 = attempt with definite intent to die.

**Table 7:** Association of depression remission (week 6), suicidal ideation remission (week 6), and lifetime suicidal behaviour with severe suicidal ideation

Predictors	OR	95% CI
Depression remission	0.64	0.31**–**1.32
SI remission	**0.43**	**0.24–0.78**
LSB score^a^	**1.26**	**1.12–1.41**
Serious LSB^a^	**2.45**	**1.29–4.66**

Significant associations (*P* < 0.025) are in bold. Depression severity (IDS-C_30_ total score) was included as a covariate.

CI, confidence intervals; IDS-C_30_, 30-item Inventory Depressive Symptomatology-Clinician Rating; LSB, lifetime suicidal behaviour; OR, odds ratio; SI, suicidal ideation.

a LSB score: from 1 = no suicidal tendency to 7 = attempt with definite intent to die.

The occurrence of serious lifetime suicidal behaviour was predicted by SI severity (OR, 2.18; 95% CI, 1.11–4.27), bipolar score (OR, 1.36; 95% CI, 1.02–1.81) and childhood sexual abuse (OR, 2.35; 95% CI, 1.09–5.05) (Table 8).

**Table 8:** Multiple logistic regression analysis of serious lifetime suicidal behaviour predictors (chi-square=25.706; df = 7; *P* = 0.0006)

Predictors	OR	95% CI
Baseline depression score	1.03	0.98–1.06
SI severity	**2.18**	**1.11–4.27**
Bipolar score	**1.36**	**1.02–1.81**
Childhood neglect	1.12	0.43–2.89
Childhood emotional abuse	0.97	0.35–2.65
Childhood physical abuse	0.88	0.34–2.25
Childhood sexual abuse	**2.35**	**1.09–5.05**

Significant predictors (*P* < 0.025) are in bold.

CI, confidence intervals; OR, odds ratio; SI, suicidal ideation.

## Discussion

This study was conducted to ascertain the clinical correlates and prognostic impact of severe SI in MDD. The importance of determining SI severity is related to its association with the dangerousness of suicidal behaviour. This relationship has been supported by an increasing body of evidence in the last few years. In prior validation analyses of the C-SSRS, subjects with the highest levels of SI at baseline had higher odds of attempting suicide during the study (Posner *et al*., 2011; Greist *et al*., 2014). More recently Shelef *et al.* (2021), administering three scales for SI to a sample of Israeli soldiers, found that greater SI severity was associated with more serious SAs even when intent to die was low. Similarly, Fernández-Montalvo *et al.* (2021), in their analysis of callers to a suicide helpline, reported that individuals with severe SI more often had preparatory acts and SAs in the last 3 months. Our results are consistent with these findings as we noted a correlation between the severity of SI during an episode of depression and the worst suicidal behaviour reported in the patient’s entire life. Hence, assessing SI remains an essential step in suicide prevention even if, as clinicians, we are aware that a substantial proportion of patients minimise or completely deny their suicidal thoughts during interviews. As suggested by a recent review, total or partial denial involves about 50% of patients with SI and 30% of patients attempting suicide (Obegi, 2021). Interestingly, among depressed patients, those who seriously attempt suicide have less willingness to self-disclose than individuals with only SI or milder SA (Apter *et al*., 2001). In other words, individuals who experience severe SI might more often conceal their thoughts in order not to be hindered in attempting suicide. Based on this consideration, knowing variables that predict severe SI could help identify individuals at greater suicidal risk and factors leading them to attempt suicide. To this end, our study emphasised the role of three potential risk factors for suicidal behaviour, such as childhood maltreatment, comorbid panic disorder and bipolar spectrum liability, as well as sleep-onset insomnia as a protective factor. The connection between childhood maltreatment and suicidality in adulthood is currently well established (Angelakis *et al.*, 2019; Xiao *et al*., 2022) and there is growing evidence that childhood maltreatment might have negative consequences for MDD in terms of SI and comorbidities (Zisook *et al.*, 2022). We further corroborated the link between childhood maltreatment and SI by demonstrating that childhood maltreatment, more common in patients who experienced severe SI, affected SI in an independent manner of depression severity. Guilt and shame could be plausible mediators of such a relationship (Bryan *et al*., 2013; Alix *et al*., 2020). Comorbid panic disorder was another predictor of SI severity, in line with previous studies suggesting a close relationship between panic and suicidal behaviour (Katz *et al*., 2011; Scheer *et al*., 2020; Zhang *et al*., 2022). A third factor connected with suicidality in our study was bipolar spectrum liability, which was found to correlate with SI severity in subjects with mild depressive symptoms. It is worth noting that childhood maltreatment and bipolar liability could predict the occurrence of severe suicidal behaviour independent of the reported degree of SI. These findings might open up intriguing scenarios for suicide prevention but further research is needed because, similarly to previous works (Fiedorowicz *et al*., 2021), we failed to demonstrate significant correlations between hypomanic symptoms and suicidality-related variables. Moreover, there might be interrelations between childhood maltreatment, bipolarity and suicidality. Childhood maltreatment is estimated to be >2.5 times more prevalent in bipolar disorder compared to nonclinical controls (Palmier-Claus *et al*., 2016) and it has been correlated with suicide behaviours in patients with bipolar disorder (Etain *et al*., 2013; Larsson *et al*., 2013). On the other hand, the presence of childhood maltreatment has been associated with SI and bipolar features in MDD (Park, 2017). Insomnia has often been pointed to as a risk factor for suicidal behaviour (McCall *et al*., 2010, 2019; Bernert *et al*., 2015; Wang *et al*., 2019; Lau *et al*., 2020; Simmons *et al*., 2020, 2021). We previously demonstrated that insomnia was a negative predictor of SI remission during antidepressant treatment (Olgiati and Serretti, 2022b). Conversely in this new analysis, sleep-onset insomnia was protective against severe SI after controlling for depression score. The finding is not easily understood in view of recent studies showing that, of all subtypes of sleep disturbance, sleep-onset insomnia had the strongest association with suicidal behaviour (Batterham *et al*., 2021). On the other hand, sleep-onset insomnia has also been related to anxiety (Bragantini *et al*., 2019), which seems to have a positive prognostic significance in depressive disorders. For instance, in the Sequenced Treatment Alternatives to Relieve Depression (STAR*D) study, patients with MDD and higher levels of anxiety were associated with more favourable SI trajectories during antidepressant treatment (Bloomfield-Clagett *et al*., 2022).

Our study analysed a large set of variables related to SI and suicidal behaviour and supported their use in clinical practice, but we are aware of a few limitations. First of all, key variables such as suicidality and childhood maltreatment were retrospectively investigated during baseline visits. This approach, besides being subject to recall bias, made it possible to ascertain suicidal tendencies before the baseline visit, but not afterward; conversely, SI and other depressive symptoms were assessed over a subsequent time span, thus their prospective impact on suicidality could not be explored. In addition, there were no scales to assess psychological correlates of suicidal behaviour such as hopelessness (Sokero *et al*., 2006; Ribeiro *et al.*, 2018), thwarted belongingness and burdensomeness (Chu *et al.*, 2017). As for bipolar spectrum variables, the ASRM scale used in this study did not include hypomanic manifestations such as racing thoughts, distractibility, and excessive involvement in risky activities and behaviours, which are often observed in mixed depression (Perugi *et al*., 2015; Brancati *et al*., 2019). In addition, family history of bipolar disorder, one of the most useful variables to distinguish bipolar II depression from MDD (Zimmerman *et al.*, 2013), was not available for our sample. Such a missing variable could have hindered the identification of bipolar spectrum cases and the assessment of their impact on suicidality.

In conclusion, the assessment of SI severity is essential during the clinical evaluation of patients with MDD to ascertain the risk of suicidal behaviour. In addition, our results provide us with other clinical insights: (1) although SI is associated with depressive symptoms, its level of severity may also be affected by nondepressive variables, such as childhood maltreatment and bipolar spectrum liability, which contribute to suicidal behaviour as well; therefore, it is recommended to thoroughly assess childhood maltreatment and bipolar spectrum features in all patients with MDD, even in those who experience milder symptoms and lower SI levels; (2) all patients with MDD who have a history of childhood maltreatment or a high number of bipolar spectrum features should be considered at increased suicide risk even if their intent to die is minimised or denied; (3) the prevention of childhood maltreatment, the provision of psychological support for individuals with a history of child maltreatment, and early recognition and treatment of bipolar spectrum features in MDD should be considered among the main targets of suicide prevention.

## Acknowledgements

The authors thank the National Institute of Mental Health (NIMH) for giving us the possibility to analyse their data on the CO-MED sample. We also thank the authors of previous publications in this dataset, and foremost, we thank the patients and their families who accepted to be enrolled in the study. Data and biomaterials were obtained from the limited access datasets distributed by the NIMH. The ClinicalTrials.gov identifier is NCT00590863.

### Conflicts of interest

P.O. received grant/research support from Italfarmaco. A.S. is or has been a consultant/speaker for Abbott, Abbvie, Angelini, AstraZeneca, Clinical Data, Boehringer, Bristol-Myers Squibb, Eli Lilly, GlaxoSmithKline, Innovapharma, Italfarmaco, Janssen, Lundbeck, Naurex, Pfizer, Polifarma, Sanofi, and Servier. For the remaining author, there is no conflicts of interest.
